# Strengthening Interprofessional Requirements Engineering Through Action Sheets: A Pilot Study

**DOI:** 10.2196/humanfactors.5364

**Published:** 2016-10-18

**Authors:** Aline Kunz, Sabrina Pohlmann, Oliver Heinze, Antje Brandner, Christina Reiß, Martina Kamradt, Joachim Szecsenyi, Dominik Ose

**Affiliations:** ^1^Department of General Practice and Health Services ResearchUniversity Hospital HeidelbergHeidelbergGermany; ^2^Department of Information Technology and Medical EngineeringUniversity Hospital HeidelbergHeidelbergGermany; ^3^Representative for people with disabilities for the City of HeidelbergHeidelbergGermany; ^4^Department of Population HealthUniversity of UtahSalt Lake City, UTUnited States

**Keywords:** personal electronic health record, requirements engineering, interprofessional cooperation, software development, scrum

## Abstract

**Background:**

The importance of information and communication technology for healthcare is steadily growing. Newly developed tools are addressing different user groups: physicians, other health care professionals, social workers, patients, and family members. Since often many different actors with different expertise and perspectives are involved in the development process it can be a challenge to integrate the user-reported requirements of those heterogeneous user groups. Nevertheless, the understanding and consideration of user requirements is the prerequisite of building a feasible technical solution. In the course of the presented project it proved to be difficult to gain clear action steps and priorities for the development process out of the primary requirements compilation. Even if a regular exchange between involved teams took place there was a lack of a common language.

**Objective:**

The objective of this paper is to show how the already existing requirements catalog was subdivided into specific, prioritized, and coherent working packages and the cooperation of multiple interprofessional teams within one development project was reorganized at the same time. In the case presented, the manner of cooperation was reorganized and a new instrument called an Action Sheet was implemented. This paper introduces the newly developed methodology which was meant to smooth the development of a user-centered software product and to restructure interprofessional cooperation.

**Methods:**

There were 10 focus groups in which views of patients with colorectal cancer, physicians, and other health care professionals were collected in order to create a requirements catalog for developing a personal electronic health record. Data were audio- and videotaped, transcribed verbatim, and thematically analyzed. Afterwards, the requirements catalog was reorganized in the form of Action Sheets which supported the interprofessional cooperation referring to the development process of a personal electronic health record for the Rhine-Neckar region.

**Results:**

In order to improve the interprofessional cooperation the idea arose to align the requirements arising from the implementation project with the method of software development applied by the technical development team. This was realized by restructuring the original requirements set in a standardized way and under continuous adjustment between both teams. As a result not only the way of displaying the user demands but also of interprofessional cooperation was steered in a new direction.

**Conclusions:**

User demands must be taken into account from the very beginning of the development process, but it is not always obvious how to bring them together with IT knowhow and knowledge of the contextual factors of the health care system. Action Sheets seem to be an effective tool for making the software development process more tangible and convertible for all connected disciplines. Furthermore, the working method turned out to support interprofessional ideas exchange.

## Introduction

In this paper, we describe how the cooperation of interprofessional teams working on the development of a personal electronic health record was reorganized in order to better integrate user requirements. As studies on computer-supported cooperative work have already revealed, successful cooperative work between different actors is a core aspect of designing products to meet the demands of future users [1,2]. In developing solutions for the health care sector, this does not only mean to take into account the multitude of future users such as patients, physicians, nurses, and so on but also to be able to cooperate when working out the characteristics of the future product. In the present case, social scientists analyzed the requirements of future users and passed relevant data on to medical information technology (IT) specialists whose task it was to implement the requirements technically.

The development of software solutions for health care is confronted with different challenges, and developer teams have to face a steadily increasing demand for IT solutions that go hand in hand with continually changing technical capabilities. Furthermore, it has become indispensable in today’s development practice to take the living and working environment of all potential future users into account [3-7]. As new and less savvy computer users receive access to online health applications such as personal electronic health records, the issue of usability becomes more critical. If newly developed software fails within the health care system because important general conditions of the care setting have not been considered, this will not only result in frustration on the part of users but in financial risks for the developing organization [8,9]. Highly qualitative requirements engineering is an essential part of efficient software development [10].

Development teams face a number of challenges connected with the effort of building up user-centered software architecture. Those challenges are located in the following areas: development tools and environment, communication and contacts, design knowledge, project management, and cultural differences [11-13]. Chen et al [14], for example, proposed an approach to bring user requirements, system design, and testing of the developed nuclear medicine software together in a 3-part model in order to meet challenges regarding development tools and environment. Communication and contacts was the second most named problem area identified by Komi-Sirviö and Tihinen [11] and was mainly connected with cultural differences and language barriers. According to the authors, efforts should be made to overcome those challenges especially by improving knowledge transfer.

An important factor of how to transfer knowledge is the way it is prepared and fed into the development process [15]. In general, there are traditional and newer ways of bringing information into the project: user-centered design (UCD) and agile software development [16,17]. While the former is characterized by a pursuit of defining the final product more or less in detail before the actual development process starts, the latter strives to build small bundles of functionalities and realize them in very short time periods [17]. It is not uncommon that both are running simultaneously within the same project context. In those cases it is important to find a way of bringing UCD and agile software development (ie, scrum) approaches together [18].

Lack of flexibility, a high amount of documentation, and little user integration are often criticized in regard to traditionally established development processes. By using agile methods, such as scrum, those problems should be overcome [19]. Scrum uses a number of roles and methods in order to systematize the software development process. The process itself is subdivided into sprint phases that last for 2 to 4 weeks. Within these time periods, prioritized features of the software are realized that were previously defined on the basis of the initial user requirements. During the sprint planning prior to a sprint, the product owner determines the tasks of the developer’s team for the next period. Afterwards the development progress is monitored within a sprint review meeting. This proceeding ensures that the developers can quickly react to changing demands and that after every sprint phase a functioning partial solution is available.

In the present case, attention was paid to the future users’ perspective right from the beginning. Thus, social scientists spent their effort on collecting data referring to the users’ opinion on what a personal electronic health record should be like and compile this information in a thorough requirements catalog. Meanwhile, a team of medical IT specialists built up the infrastructure and user interfaces of the future product by using the scrum approach. In the course of the project it became clear that the proceeding of the medical informatics site was quite opaque for all those who were not directly connected with the technical development. In addition, the social scientists had problems conveying the data obtained from user surveys to the medical IT specialists.

The following research question was the basis of the described methodology development: How can user-reported requirements be better integrated into the software development process?

This paper presents the results of a newly developed methodology of bringing those two approaches together in order to smooth the development of a user-centered software product and to restructure interprofessional cooperation.

## Methods

### Overview

A pilot project called Information Technology for Patient-Centered Health Care (INFOPAT), funded by the German Federal Ministry of Education and Research (2012-2016), has been initiated in the Rhine-Neckar region [20]. This project aims to improve care across different health care settings especially for chronically ill patients. A central component for reaching this aim is the development of a patient-controlled electronic health record (PEPA) [21,22]. The PEPA endeavor is divided into a technical research and development project and an implementation project. The first deals with the concept, design, and implementation of the PEPA’s system architecture and its components as well as integration aspects. The latter focuses on the composition of user requirements and on the challenges of PEPA implementation into the care process of colorectal cancer patients. The study was approved by the Ethics Committee of the University Hospital Heidelberg (S-497-2012). All participants gave their written informed consent. Participant anonymity and confidentiality was ensured throughout the study.

Within the first project phase of the development project, the PEPA infrastructure has been developed and implemented by a team of scientific and industrial partners [23]. At the same time, a requirements catalog was compiled by members of the implementation project [21,24]. The foundation of this profile has been laid by performing focus groups with patients, physicians, and other health care professionals (HCPs). One goal of the INFOPAT project was to gain wide-ranging knowledge on colorectal cancer care. The complexity of this illness and the cross-sectoral health care setting might be positively influenced by a more active patient role according to managing their illness with the help of information and communication technologies (ICTs). Therefore, 10 semistructured focus groups (N=47) were conducted with patients diagnosed with colorectal cancer, representatives from patient support groups, and physicians and other HCPs in the Rhine-Neckar region in order to gain knowledge on the participants’ experiences regarding colorectal cancer care and their attitude referring to the PEPA concept [24]. Patients were recruited through the National Center for Tumor Diseases (NCT) in Heidelberg, Germany, and an umbrella organization for patient support groups in Heidelberg. Physicians and nonphysician HCPs (eg, nurses, stoma therapists, social workers, physiotherapists, and nutritionists) were either involved in colorectal cancer care at the NCT or in the ambulatory setting (general practitioners, oncologists) All focus group meetings were audio- and videotaped, transcribed verbatim, and thematically analyzed using qualitative content analysis. Based on these data, a systematized and prioritized version of the requirements catalog containing 245 user demands was developed which then could be realized within the already existing PEPA infrastructure [21]. For this development step, a close feedback cycle between both teams (medical IT experts and social scientists) is mandatory [25]. In the course of the project it proved to be difficult to gain clear action steps and priorities for the development process out of the requirements catalog. Even if a regular exchange between both teams took place there was a lack of common language. Therefore, the following steps were conducted with the result that all partners agreed to use the resulting instrument, called Action Sheet, with the objective of improving the interproject cooperation.

### Problem Analysis

In order to identify general problems of information exchange between the development project and the implementation project part, several interprofessional meetings took place. A list of identified weaknesses was created that included, for example, missing acceptance criteria, a need for specific user stories, or too little involvement of already elaborated concepts of PEPA functionalities.

### Review of Literature

To identify papers referring to the specific problem contexts, a literature search was carried out. The main goal of this search process was to gain insights into the management of user requirements within other projects especially referring to development of health IT solutions. The review was performed by social scientists working in the application team. The literature sources mainly had a strong focus on the medical informatics perspective. Therefore, it was hard to gain comprehensive knowledge on possible solution approaches within a short period of time. Nevertheless, a number of common practices were recommended by the development project team that appeared to be helpful for overcoming the existing challenges.

### Interprofessional Discussion

After receiving general knowledge of different possibilities for managing user requirements, both teams agreed on those which seemed to be most relevant and applicable for the current project context. On this basis the structure of Action Sheets and necessary categories was drafted. The draft versions of the structure and scopes of Action Sheets were discussed in two interproject meetings. Both teams agreed on the mandatory structure of the instrument for the forthcoming project steps. Furthermore, the scopes were prioritized regarding their necessity for the upcoming milestones like, for example, provision of usability tests with patients or professionals.

## Results

### Formalization of User Requirements

Until the decision for restructuring interprofessional cooperation was made, the implementation project team provided a more epic kind of requirements catalog. One user requirement, for example, was summed up by a sentence like “The PEPA has to provide the opportunity to upload data manually.“ Along with this statement an explanatory paragraph was handed over to the development team in order to enter it into the scrum-based development process. In order to improve the interprofessional cooperation the idea came up to align the requirements arising from the implementation project with the scrum approach of the technical development team. Therefore, based on intensive interprofessional discussion, a number of formalization techniques was agreed on between both teams that should be used in the ongoing steps of usability design of the PEPA. The following development methods were chosen for restructuring the already existing requirements catalog of the social scientists:

- Scenarios
- Sketches
- User stories
- Acceptance criteria

Those methods are used in most UCD projects but were not worked out for the project context so far because the original development was based on the mentioned requirements catalog. Furthermore, the team agreed on a series of additional categories for communicating a feeling for how the PEPA should be like from the user’s point of view that is as tangible as possible. Those categories and their fusion in the form of Action Sheets will be described in the following paragraphs.

### The Idea of Action Sheets

**Figure 1:**
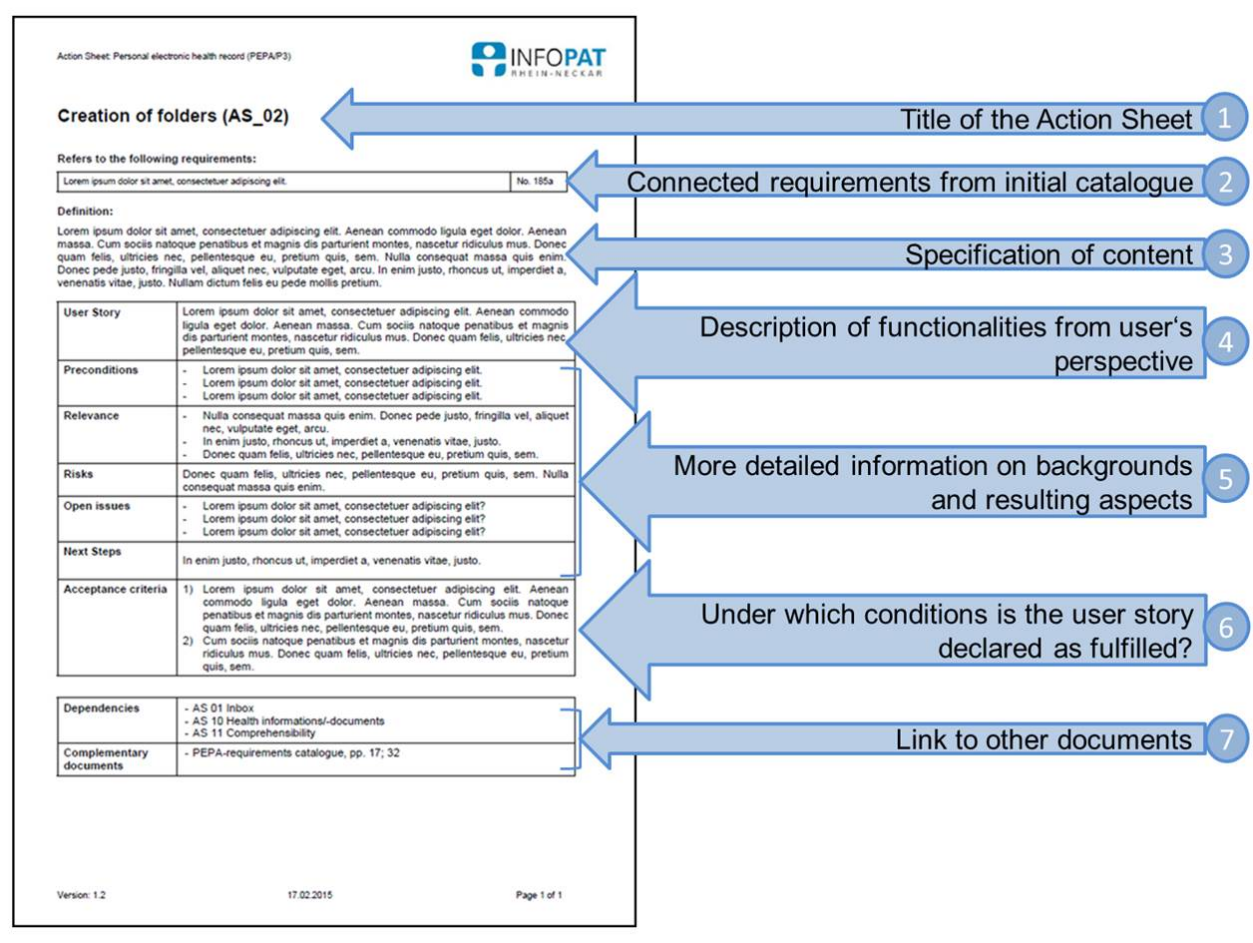
Standardized structure of Action Sheets.

Since the new instrument was meant to push the interprofessional work, the name of the newly developed working papers was more or less obvious-Action Sheets were born. In a first step the implementation project members provided a set of 26 Action Sheets that were discussed with the development team in order to make sure that the content was aligned according to all requirements whether arising from technical feasibility or user demands. Generally, Action Sheets are meant to illustrate the way future users want the product to be in a standardized manner. Therefore, they delve more or less deeply into the different aspects of characteristics and functionalities but without getting lost in the details. The initial PEPA requirements catalog, for example, contains 245 user demands that were gained from the focus group discussions. Those demands were restructured and prioritized and form the basis of Action Sheets. Every Action Sheet follows the same layout structure and consists of the same elements (see Figure 1).

All PEPA Action Sheets should serve as specific example cases that help to advance the prototype functionalities in a targeted manner. In order to attain this objective, every Action Sheet consists of 14 categories but not all of them are mandatory for each requirement set (see Table 1). The title, connected requirements from the initial catalog, and a definition are part of every Action Sheet. With the elaboration of the catalog, a paper-based mock-up of a possible conceptual implementation of user requirements was designed as well. Parts of this document are integrated into the Action Sheets as sketches whenever possible in order to demonstrate a thinkable logical structure of the health record and its user interface.

**Table 1:** General content of Action Sheets.

No.	Category name	Content/meaning
1	Title	Designation of connected PEPA features or characteristics
2	Connected requirements	Most important aspects/user demands
3	Definition	Short description of the purpose of the Action Sheet and its importance for the PEPA concept
4	Sketches (if applicable)	Images of design proposals for PEPA features if applicable
5	User Story	Short and precise summary of user demands addressing the questions “Who wants what?” and “What is the aim?”
6	Preconditions	Prerequisites that must be met in order to make the user story come true
7	Relevance	Facts added to the explanatory statement briefly mentioned within the user story
8	Risks	Factors that might endanger a successful realization of the Action Sheet
9	Open issues	Uncertainties or contradictions that must be clarified for a successful practical application
10	Next steps	Activities that must be carried out for successful realization of the respective requirement
11	Acceptance criteria	Ways to test if the user story can be declared as fulfilled
12	Dependencies	Direct relations between different working papers
13	Complementary documents	Other possible references
14	Attachments	Other supplementary material

A necessary component of Action Sheets is the user story, which contains a short and precise summary of user demands covered by the respective working paper. According to the scrum method, the user story should encourage the developers to think of their work from the perspective of who will use it using tangible examples. Typically the user stories should be structured in a certain way following the questions “Who *[end user]* wants what *[requirement]*? What is the aim *[explanatory statement]*?”.

The next category is Preconditions and as the name suggests it covers necessary prerequisites that must be met in order to make the user story come true. The Relevance category highlights the importance of the issues that are summed up in a Action Sheet. This category offers space to add facts to the explanatory statement that is briefly mentioned within the user story. If a successful realization of the requirements covered by an Action Sheet may be in danger because of certain surrounding conditions, those conditions will be listed in the Risks paragraph. The Open Issues section provides the opportunity to make ambiguities or contradictory aspects a subject of interprofessional discussion. User-centered software development is a complex, iterative process, so every emerging step of development depends on related topics that have to be clarified beforehand. Those topics are registered under Next Steps.

The Acceptance Criteria is a measure to gauge whether all requirements covered by the user story are met or not. Similar to the user story it is geared to the scrum method and follows a fixed structure consisting of prerequisite, action, and result: assumed [...], if [...], then [...].

Since many requirements that have been summed up in Action Sheets are interdependent, the category Dependencies shows direct relations between the different working papers. Under Complementary Documents a link to the initial requirements catalog as source of user demands and to important other resources will be created. As some of the initial requirements are redundant and some others are not that relevant for the overall context, the Connected Requirements paragraph at the head of each Action Sheet only consists of the most important user demands. Other connected issues are summed up at the bottom section called Attachments. Once the components and overall structure of Action Sheets was agreed upon between the development and the implementation project teams, it was a challenge to decide for which subject areas particular Action Sheets are needed. After a number of central issues (eg, graphical user interface, data security, emergency access, and creation of folders) were defined from the entirety of requirements, the associated requirements were subordinated. Based on the central issues, the first version of Action Sheets was set up with an iterative design and became the origin of the ongoing development process.

**Figure 2:**
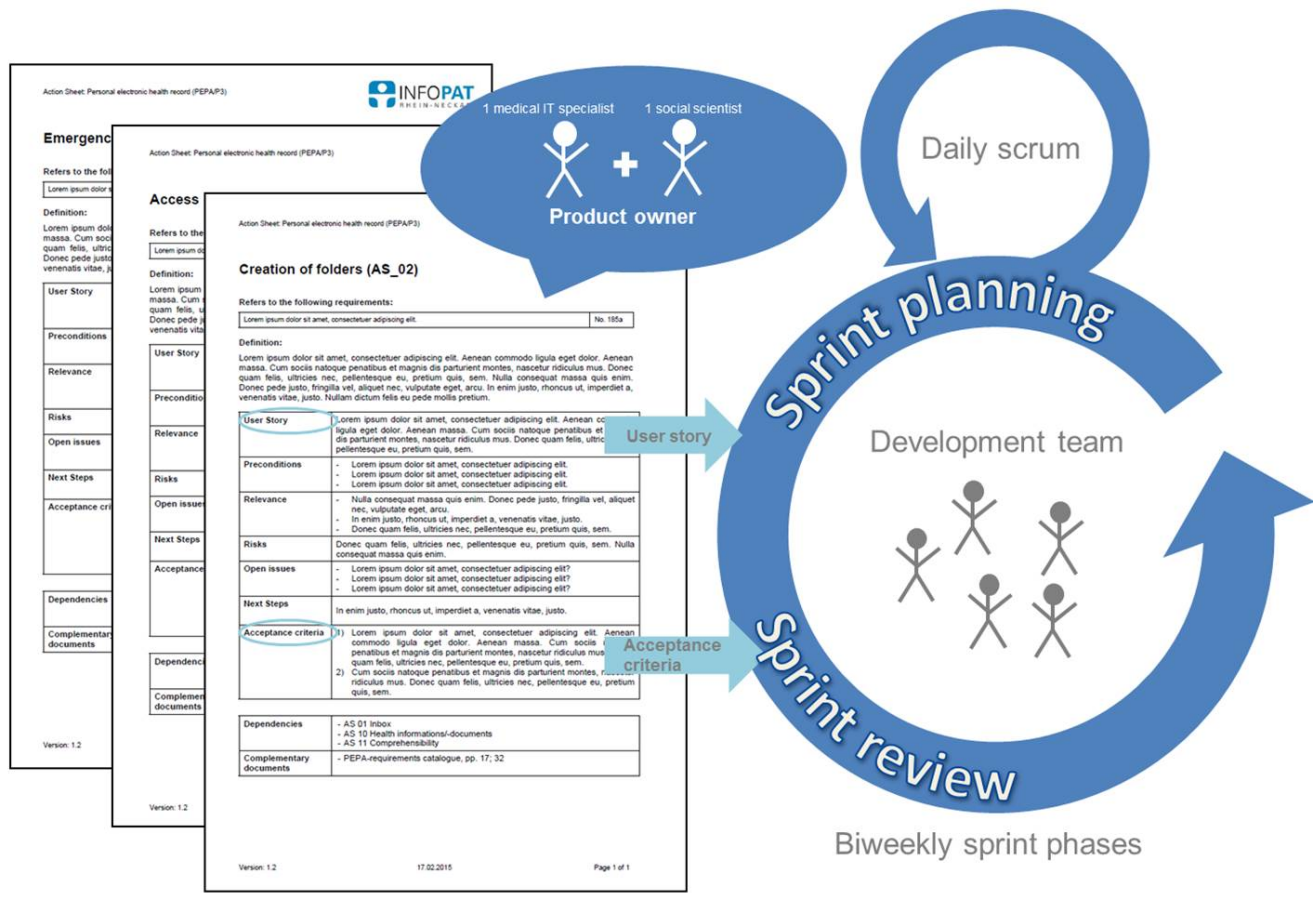
Action Sheet-based feedback cycle.

### Integration of Action Sheets Into the Development Process of the PEPA

As already mentioned, the scrum approach applied by the technical development team was adapted. As a slight modification of the scrum working manner, the role of the product owner became split up into two responsible persons, one medical IT specialist from the development project and one social scientist from the implementation project. Those two persons take responsibility for the consolidation of guidelines for the PEPA development by creating, rating, and explaining the product properties that should be built up to the developers. An additional central aspect of this role is to decide which steps should be taken during the 2-week sprint phases.

The product owner team agrees on the content of the Action Sheets and on the order of bringing those requirements into the sprint process (see Figure 2). Thus, both persons responsible for the product owner role take the Action Sheets as groundwork for discussing the actual action fields with the developer team within the sprint planning and the sprint review meetings. Based on these discussions, the 2-week sprint phases are scheduled according to previously set priorities and with regard to the tasks of the preceding sprint phase that were not yet solved to satisfaction. At the start of every new sprint phase, the user stories of the Action Sheets serve as input for the sprint planning because they set a clear frame of functionalities that have to be fulfilled in order to let the respective user story be realized. The acceptance criteria helps to test if the user story can be declared as fulfilled within the sprint review meeting at the end of every 2-week period.

### Experiences With Action Sheet–Based Cooperation

The first feedback from the development and the implementation project members indicates that it is easier for all persons involved to capture other points of view and mindsets. One example for this finding is the first Action Sheets–based discussion referring to the topic *inbox*. According to user requirements an Action Sheet was created that summed up available and relevant information on an inbox within the PEPA. Patients participating in the focus groups stated that they would like to be informed about new entries and documents in their PEPA, similar to the inbox of their email account. For this reason, a first meeting was arranged between social scientists knowing about the future user idea of this inbox and medical IT specialists knowing about the technical feasibility. The Action Sheet helped to follow a structured procedure in order to discuss all relevant factors referring to this topic. Social scientists learned that an inbox that worked exactly the way patients wanted it to be would be unrealizable because of technical circumstances. In addition, medical IT specialists understood why it was so important for patients to have the possibility to build up their own folder structure in order to systematize documents within their PEPA according to their own logic. Based on this joint brainstorming, the Action Sheet was revised so that it could serve as guideline for continuing development steps.

**Advantages and challenges of Action Sheet–based cooperation.**

Advantages

- Clearer understanding of user demands
- Close supervision of project progress
- Quick identification of divergent interpretations
- Easier achievement of common wording

Challenges

- Expenditure of time for elaboration of Action Sheets
- High administrative effort for Action Sheet fundamentals
- Time-consuming, closer interdivisional collaboration
- Accidental omission of important features

In total, 14 team members were closely involved in the Action Sheets–based work. For most of them, the introduction of Action Sheets was associated with a higher expenditure of time for a renewed processing of the requirements catalog. Additionally, time was needed for more frequent interprofessional consultations and appointments. It took about 3 months of close interprofessional coordination until the first version of Action Sheets was compiled.

However, the discussion on important aspects of the development process became much more focused, and the expectation of the project partners was promoted. Textbox 1 gives an overview of advantages and challenges arising from the Action Sheet–based operating principles identified by all team members.

Even if the entire requirements catalog was used as basis for the Action Sheet development and a relative large scale of knowledge is transported via this measure, it is still possible that important features could accidentally not be taken into account or not yet completely be integrated into the working process. However, it only became possible to unfold a common understanding and wording because of the focused and detailed Action Sheet–based debate about what needs to be done within the development progress. Divergent interpretations of user requirements were identified quickly so that a common consensus could be achieved.

Of course, this debate was associated with quite an expenditure of time for the elaboration of Action Sheets. But hand in hand with this time effort went the fact that a really close supervision of the integration of user demands into the development of underlying user stories was enabled. This was mainly influenced by the closer interindividual collaboration that was put into practice by realizing a number of team meetings. For example there were a lot of out-of-turn ballot meetings taking place in addition to the biweekly sprint review meetings held in the presence of at least one member of the implementation project team. The most striking challenge that had to be faced when implementing the Action Sheet workflow was the high administrative effort for building up the first Action Sheet fundamentals. It was a complex process to work out a standardized Action Sheet prototype, to make a first draft of all necessary Action Sheets and, last but not least, to adjust all of them in interprofessional collaboration. Still, this was a good way to get a clear understanding of user demands which is the most central aspect of the PEPA concept.

## Discussion

### Principal Findings

This research project examined how user-reported requirements can be integrated into the agile software development process based on scrum in a better way. In the present project context it was a challenge that requirements named by future users sometimes did not meet the theoretical understanding of IT specialists or rather the technical realizability in the first place. Therefore, both teams made up their minds to introduce an approach that enables a common, iterative development process of the PEPA prototype. It was a broad-based consensus that the social scientists and the medical IT perspectives had to be joined and circumscribed in a way that is generally intelligible. For that reason, both sides agreed that it would be beneficial to elaborate a working base that is matched to the respective viewpoints.

In this context, it proved to be helpful to make use of an instrument called Action Sheets in order to integrate the user requirements more directly into the development process. Action Sheets serve as a communication bridge between different methodological approaches, enable a more standardized action, and were implemented as a commonly accepted working base. Bossen et al referred to boundary objects in a similar context [26]. They stated that for developing and implementing health IT solutions persons without medical expertise should be included into the design processes. In their example the role of medical secretaries in a hospital setting for successful implementation of an electronic health record was shown [26]. According to Star and Griesemer’s (1989) concept of boundary objects [27,28], Bossen et al say that “coordination mechanism can become boundary objects that facilitate and stabilize cooperation between different social worlds, whose actors relate differently to but cooperate through these” [26]. Action Sheets as vehicles for combining the social scientific viewpoint and the medical IT perspective in the present case therefore also could be seen as a kind of boundary objects.

The necessity to uncover dependencies of software functionalities as early as possible within the development process is already known from other studies [29]. The reason is that only on the basis of a clear picture of user requirements a prioritization of development tasks can be carried out. But it is also known that those dependencies of functionalities and general user requirements can be addressed in different methodological ways: UCD or agile development concepts are examples. In practice, those methods often collide when used at the same time by different organizations of the same development context [17]. Nevertheless, experience shows that a combination of UCD and agile development within the same project also holds benefits for the final product as far as different preconditions are fulfilled [18]. Inayat et al name user participation, team structure, and communication culture as important prior conditions of successful combination of both working methods [18].

Another essential success factor in development processes is efficient knowledge management. Whenever players who often do not share the same professional background are part of a development process, misunderstandings and uncertainties can occur. These tend to influence the quality of the development itself and therefore of the resulting product [11]. Chen et al presented their approach for overcoming this obstacle which covers the whole product development process from managing user requirements to testing of a final product [14]. However, Action Sheets need to be differentiated from this approach since they are not that far-reaching. They rather could serve to support working on the first (requirements analysis and project planning) and maybe the second (solution exploration and system design) milestone mentioned by the authors.

A further core element of accomplishing integration of user demands especially into agile methods is continuous validation of collaboration patterns [10,30]. In order to generate good performance it is essential to communicate about the thinking of other team members. For that reason, it sometimes can be beneficial to reorganize teamwork, for example, to enable the breaking up of established ways of communication or uniform thinking patterns [31,32].

An Action Sheet–based approach addresses exactly the above mentioned aspects. It helps to set a new basis for collaboration and breaks through previous communication and agreement procedures. Additionally, it creates a more tailored picture of user requirements because of systematic reorganization of knowledge on demands and technical feasibility. Generally spoken, the Action Sheets approach is an instrument that supports the merging of agile working methods and UCD.

Taking into account the growing importance of ICT in health care, it can be assumed that instruments like this gain importance as well. Whenever something is meant for usage in a health care setting it is more or less clear that different ideas of the product of software development must be merged in consideration of technical aspects. Therefore, it is indispensable to take the user demands into account from the very beginning and bring them together with IT know-how and knowledge of the contextual factors of the health care system. Action Sheets seem to be an effective tool for making the software development process more tangible and convertible. The working method supported interprofessional ideas exchange and helped to reveal areas of the concept or the prototype that need further discussion on how to realize the user’s image of the future product.

### Strengths and Limitations

Because the development of the PEPA is still going on, it cannot be concluded that the modified working method will have sustainable positive impact on the process of interprofessional cooperation. Still, for the ongoing PEPA development process it was very helpful to see the project context in a new light by the help of Action Sheet implementation.

With the repeated dealing with the content of the requirements catalog the possibility arose to put the emphasis of PEPA development in more concrete terms. This had positive impact on the project progress because a more focused and uniform destination route was exposed. Still, the split product owner role might lead to situations where it is not clear who has the final decision-making power. This weak spot of the concept should be overcome by a generally accepted solution for concrete disputes, for example, by involving the steering board of the overall project. Furthermore, it should not be concealed that the development of Action Sheets was connected with a high amount of administrative efforts as mentioned above. These efforts do not meet the scrum demand for little documentation in the first place [19]. But, if Action Sheets would have been integrated into the development process earlier this double effort would not have been necessary. Therefore, it can be recommended to implement the Action Sheet instrument before the development process actually starts.

### Implications for Research Practice

As part of the second project phase, the operable PEPA prototype will be evaluated within the real care setting of cancer patients. Therefore, usability tests and interviews with patients treated for gastrointestinal tumors, patient family members, and health care professionals will be conducted in a first step. Afterwards the prototype will be customized according to user feedback and then brought into test use for 3 months. Within this test phase, patients suffering from colorectal cancer and being treated at the NCT in Heidelberg will get the opportunity to use the prototype for preparation of and follow-up of their regular medical appointments within the center and in family practice. These evaluations will lead to further feedback necessities between both project teams. If the Action Sheet–based cooperation proves beneficial, it will be possible to revert to an already established working method within the second project phase. The user feedback could be reflected immediately so that the optimization of the prototype will hopefully work more quickly and smoothly.

All persons involved should be asked for their assessment regarding the initiated Action Sheets working manner. Therefore, a structured user evaluation is planned after the development project has ended. The results will help to further improve the application of Action Sheets as working method, and more information can be obtained on how to structure interprofessional cooperation in a development process right from the beginning.

## Abbreviations

HCP: health care professional
ICT: information and communication technologies
IT: information technology
INFOPAT: Information Technology for Patient-Centered Health Care
NCT: National Center for Tumor Diseases
PEPA: patient-controlled electronic health record
UCD: user-centered design
